# Nepal Health Conclave 2024: Coordinating Diaspora for Healthcare Strengthening

**DOI:** 10.31729/jnma.8886

**Published:** 2025-02-28

**Authors:** Madhusudan Subedi

**Affiliations:** 1 Department of Community Health Sciences, Patan Academy of Health Sciences, Lagankhel, Lalitpur, Nepal

## INTRODUCTION

The contributions of diaspora members have been recognized by the Government of Nepal by granting legal status and enabling them to work and invest in Nepal. In order to strengthen the health care and services of the country, the Ministry of Health and Population (MoHP) hosted a Nepal Health Conclave in Kathmandu on December 26-27, 2024. The theme of the Conclave was "Nepal Health Conclave 2024: Coordinating Diaspora for Healthcare Strengthening". The specific objectives of the event were:

1. Explore how the diaspora can support Nepal's healthcare through expertise, resources, and partnerships.
2. Promote knowledge and technology exchange between the Nepalese Medical diaspora and local healthcare providers.
3. Promote collaboration in research innovation and training to help Nepali researchers access global funding and resources.
4. Identify policy factors affecting diaspora engagement in health and create strategies for sustainable partnership.

The conference focused on fostering partnerships, sharing knowledge, and addressing the challenges facing Nepal's health sector by mobilizing the knowledge, skills and resources of Nepali diaspora health professionals and local health / public health workers. It was said by the spokesperson of the MoHP that 1,000 registered from 90 countries among them 300 participated in a face-to-face mode while 700 attended virtually. There were high level discussions on these four thematic areas into ten dedicated sessions.^1^

The participants highlighted and expected that the conference would promote knowledge and technology exchange between the Nepalese medical diaspora and local healthcare providers. The major focuses were on

the engagement of diaspora on 'e-health' and advanced health, policy dialogue and long-term engagement and research, education and innovation and the Nepal Medical Diaspora Engagement Framework.^2^

## TEN KEY PRIORITY ACTIONS

The conclave declared in the adoption of 10 key priority actions aimed at strengthening the country's healthcare systems.^2^

1. Establish Research, Evidence Synthesis and Diaspora Engagement Unit at MoHP, backed up by Diaspora Engagement Advisory Team.
2. Integrate Diaspora Engagement Activities into National Joint Annual Review (NJAR).
3. Establish an Idea/Project Bank as a centralized platform to address Nepal's health sector needs, promoting collaboration with startups and driving investment in the health sector.
4. Develop, maintain, and extend diaspora mapping/ horizon (Who is Who? Where?).
5. Design Virtual Knowledge Platforms for continuous learning, mentoring, and capacity building.
6. Establish a platform for Diaspora Research Grants to fund priority and under-represented health issues.
7. Explore possibilities to engage diaspora to address health issues of Labor Migrants.
8. Pilot innovative diagnostic and critical care services through diaspora engagement.
9. Develop and functionalize Rural Health Fellowship Programs for diaspora.
10. Conduct policy analysis to identify the policy and legal arrangements required for improved diaspora engagement in health sector investment.

The outcomes of the conclave are expected to further facilitate the formulation of strategies for long-term collaboration between local and international doctors, health workers and public health workers and draw actionable policy recommendations to strengthen the healthcare system.

## CRITICAL REFLECTION AND WAYS FORWARD

The ten key priority actions made during health conclave are highly ambitious but not impossible if the diasporas are really committed to improving public health systems and outcomes in Nepal. Clear actionable priory agenda are needed to implement and sustain these priority actions. An effective implementation requires robust frameworks of each priority action that outline timelines, resource allocation, leadership and management, and accountability mechanisms and these issues should be explored within each thematic group of stakeholders in Nepal and abroad.

Without the involvement of universities, academy and research institutions, the Research Synthesis and Diaspora Engagement Unit at the MoHP would not be sustained. A conducive social and political environment for diaspora in Nepal, along with their commitment to investing in and supporting the overall health system, is necessary to turn this dream into reality.

Furthermore, the ambition for interim popularity can lead to over-optimistic promises. Leaders and stakeholders should focus on showcasing impressive initiatives to gain political or public favor, and sustained efforts are required for meaningful change and genuine progress. Moreover, it risks misallocating resources due to political influences that lack strategic depth, sidelining more pressing but less glamorous issues. To transform these events from symbolic gestures into catalysts for change, a balance must be struck between ambition and realism, reinforced by transparent strategies and a commitment to sustained action. It is important to translate the commitments into actionable steps, ensuring that the strategies and pledges made during the conclave are effectively implemented to create tangible improvements in Nepal's healthcare sector. This holistic approach should be developed to enhance the quality of healthcare services and build a more resilient and selfsufficient health system for the future.

The conclave should be continued, implementation framework should be widely discussed with the relevant stakeholders, and success and failure stories should be documented and updated regularly.
